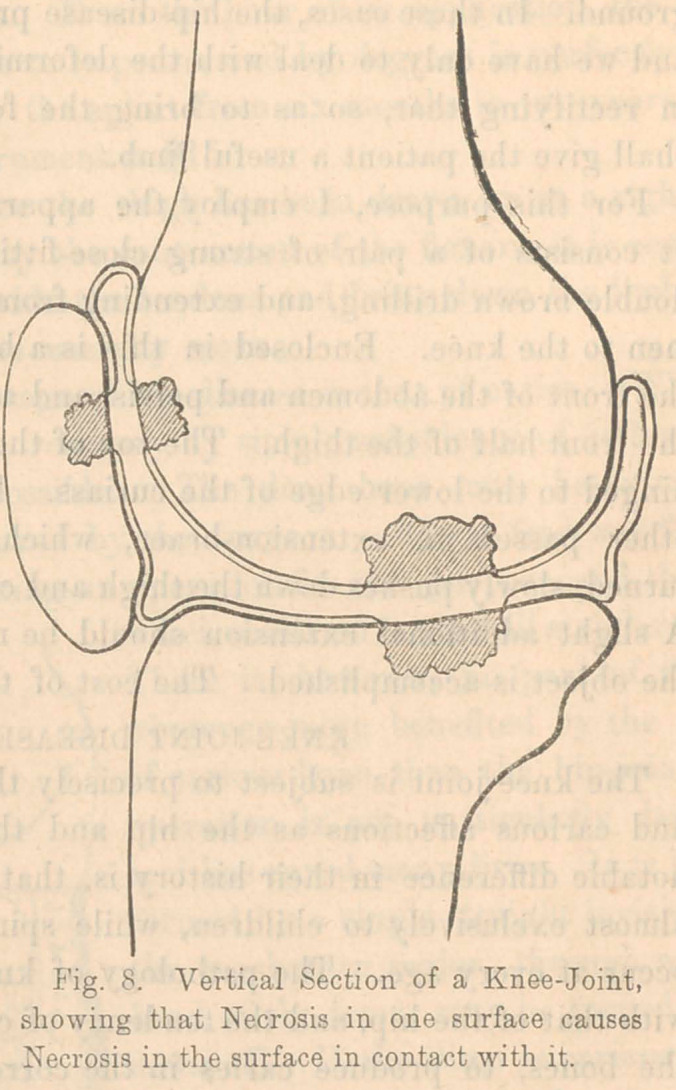# Improved Methods of Treatment in Joint and Spinal Diseases

**Published:** 1863-09

**Authors:** E. Andrews

**Affiliations:** Prof. of Surgery in the Chicago Medical College


### CHICAGO MEDICAL EXAMINER.

N. S. DAVIS, M.D., Editor.

VOL. IV. SEPTEMBER, 1863. NO. 9.

### Original Contributions.

ARTICLE XXII.

### IMPROVED METHODS OF TREATMENT IN JOINT AND SPINAL DISEASES.

By E. ANDREWS, M.D., Prof. of Surgery in the Chicago Medical College.

Very important improvements have of late been made in the treatment of diseases of the spine, the hip, the knee, and the ankle. So rapid and brilliant has been the advance in this department that, among the best surgeons, the treatment of joint and spinal diseases is already revolutionized, and vast numbers of cases are now easily curable, which formerly defied our utmost skill. It is to be regretted, however, that very many of these improvements are still unknown to the great mass of our profession. Even some of our most recent text-books are behind the times, and repeat the advice of ten years ago, in utter unconsciousness of any recent progress having been made.

As the information has never been given to the public in any compact and comprehensive form, there are many practitioners, who, though aware of the existence of such improvements, have not been able to obtain sufficient knowledge of their details to apply them in the treatment of their patients. The mechanical difficulties to be overcome are considerable, for the apparatus cannot be purchased ready made, but must almost always be constructed by measure to fit the patient, making, of course, a constant tax upon the ingenuity of the surgeon; besides, the medical man, out of the cities, often has no mechanic at his command, with the skill required to execute his plans.

For these reasons, it happens that the whole country is sprinkled with cases of neglected deformity and articular disease, which have never been taken seriously under treatment, and many of which are susceptible of complete cure.

The object of the present article is, to remedy this deficiency, by giving a carefully condensed and illustrated synopsis of the best modes of treatment now known. I hope to make the essential points so clear that any surgeon, who has ingenuity and access to suitable mechanics, will be able to devise and have constructed everything which the treatment requires; or if he does not wish to enter upon this branch of practice, he will, at least, be made aware of the numerous cases which are now proved to be curable, and can direct the patient to some one who will take him efficiently in charge.

It being no part of my object to make a special parade of my own improvements in this branch of surgery, nor to settle the disputed authorship of those made by others, I may be excused from all controversial remarks. Suffice it to say, that the advances which have been made are the joint offspring of a number of different minds on both sides of the Atlantic. Probably, Dr. Henry Davis, of New York, is entitled to more credit than any other one man, for the impulse which has been given to this progress.

The diseases to which the late improvements are mainly applicable are the following:—

Curvature of the Spine, (Spinal Disease,)
Hip Disease, (Morbus Coxarius,)
Knee-Disease, (Inflammation and Caries,)
Club-Foot, (Talipes.)

Our brief space forbids any more extended remarks upon the pathology of these diseases than will suffice to show the principle on which the treatment rests.

### SPINAL DISEASE.

Spinal distortions result from inflammation, caries, rickets, chronic contraction of muscles, paralysis, wrong habits of position in study or work, and unequal development of the muscles on the two sides by the exclusive use of one set, as, for instance, in sewing girls. Inflammation and caries usually produce the backward distortion; and the angular variety, while the remaining causes result in the lateral deformity and the curved forms. Lateral curvature, is almost always double, like the letter S.

*Constitutional Treatment.*—The most important modern improvements are of a local and mechanical character, but the correction of the general health must not on that account be overlooked. Thus, if paralysis, rickets, scrofula, or any other disturbance is present, the well-known standard remedies are to be used. Some special remarks, however, are required respecting the correction of the diathesis in inflammatory cases. Inflammatory spinal disease may be divided into two stages,—first, that of simple inflammation; and the second, that of suppuration and caries. If the patient is of a very plastic diathesis, suppuration and caries occur with difficulty, if at all, and an excellent opportunity is afforded to effect a perfect cure. If, on the contrary, the diathesis at any time becomes aplastic, the inflamed vertebræ may become carious at once, after which, the life of the patient is in extreme peril. It is of the utmost consequence, therefore, to maintain a uniformly plastic diathesis by proper constitutional treatment.

For the preservation or restoration of plasticity there is no medicine practically equal to the perchloride of iron. This should be given in doses of 20 to 40 drops of the muriated tincture, for an adult, every three hours. Quinine and mineral acids are also extremely valuable.

The diet should consist largely of meat, and be in all respects rich and nutritious. The patient should also spend much time out of doors, and at night sleep where every breath inhaled will be of perfectly pure and fresh air. By acting thoroughly upon these principles the diathesis can usually be rendered and kept perfectly plastic, and, if this is accomplished, caries will rarely supervene.

In all inflammatory affections of the joints, the pressure of the weight of the body upon the diseased articulation is a most exasperating and injurious element in the disease. It is for this reason that the lower half of the spine, and the joints of the lower extremities far more frequently run on to destructive suppuration and caries than the upper. One of the most important discoveries ever made, therefore, is the recent one, that in treating diseases of this class, *the weight of the body must be taken off, and the tension of the muscles must be overcome, so that all pressure shall be removed from the affected articulation.* The

Fig. 1. Hip Armor and Adhesive-Strap Jacket.

mechanical difficulties in the way of accomplishing this end, in diseases of the spine, have been very great, but by patient ingenuity they are now, in a great measure, overcome.

If the disease is inflammatory and is not higher than the sixth dorsal vertebræ, I make use of what, for want of a better name, I may call the hip armor and adhesive-strap jacket, which is constructed in the following manner:—(See Fig. 1.) First, take a complete cast of the patient's hips in plaster of Paris, from the small of the waist downward to two inches below the *trochanter major.* Using the cast as a pattern, have a brass armor hammered to fit it, making it wide on each side, somewhat narrower behind, and still more narrower in front, so that the thighs may not press against the lower edge when flexed.

This armor opens by hinges situated a little external to the sacro-iliac junctions and locks in front on the linea alba. It is, therefore, composed of three pieces; and, when clasped upon the patient, will be found to fit the hips nicely, and to bear any amount of downward pressure, without causing pain. It should be lined with cotton flannel. A steel rod arises from the centre of the back of the armor and another from the front, each coming well up to the height of the shoulders. Their upper extremities are cut for eight inches into a screw, and carry an octagonal nut. A short and strong jacket must be made to fit the chest, closing snugly with buttons under each axilla, and fastened at the top to the circumference of a steel ring which surrounds the neck. This ring has sockets before and behind, which slide down upon the screws to a distance regulated by the nuts. The centre of the jacket, at the back and front, is made of "elastic," (similar to the "gores" of Congress gaiters,) to keep the garment always snugly drawn against the skin, and, at the same time, to allow of the motions of the ribs in respiration. When this is finished and ascertained to fit firmly and closely, it is to be lined with adhesive-plaster throughout the inside, except where the elastics come. The adhesive-plaster must be securely sewed on, especially at the upper border. Finally, a strip of elastic webbing, carrying a pad covered with oiled silk, is to be attached on either side of the ring behind, passed under the axilla, and buckled to the ring again in front as tightly as may be found necessary.

If now the nuts on the two screws be turned upwards, the ring will be raised, and, by the tension upon the adhesive-plaster and upon the axillary band, the weight of the upper half of the body may be entirely taken off from the spinal column and borne by the steel rods directly upon the armor of the hips. The source of irritation being thus removed, the inflammation will, in many instances, subside spontaneously without any other treatment. At the same time, the spinal column is drawn straight, exactly as if it were a string. The adhesive-plaster should be renewed once in two weeks, and the skin under it thoroughly washed. The cost of the apparatus is about $25.

A simpler apparatus will accomplish the end desired in cases which are not inflammatory, because, in such instances it is not necessary to take off the weight of the trunk, but only to straighten it. Where a non-inflammatory curvature is lateral, which it usually is, I advise the instrument shown in Fig. 2. This consists of a wide band of strong drilling, fitted closely to the form of the hips, enclosing a large brass plate in the back and another in front. From each of these plates rises a flat steel rod to the heighth of the seventh cervical vertebræ behind, and of

Fig. 2. Instrument for Double Lateral Curvature without inflammation.

the top of the steruum in front. A half jacket connects the summits of the two rods passing around the shoulder on the same side as the concavity of the upper curvature. A broad "elastic" passes around the opposite side lower down, so as to press firmly upon the convexity of the upper curve. On the opposite side from this, still lower down, passes a second elastic which presses upon the convexity of the lower curve. Both elastics should buckle to the front rod in such a way as to allow of strong tightening. This apparatus is extremely light, simple, and efficient. When desirable, the principle of Fig. 1 and Fig. 2 may be combined, by attaching the elastics to the rods of the former. The apparatus of Fig. 2 costs about $15.

When the distortion is backwards, an entirely different instrument is required. If inflammation still exists, apparatus No. 1 should be applied, but if that stage is past and the deformity alone is to be treated, it can be admirably managed by the principle represented in Fig. 3. It consists of a steel spring enclosing the hips, shaped like those of trusses and attached to a brass curiass in front, which distributes their pressure upon the lower half of the abdomen, and the front of the pelvis. From the back of the spring rise two strong elastic steel straps, set wide enough asunder to avoid pressing on the bony prominences of the deformity, and to make pressure upon the common mass of the *erector spinæ* muscles on either side. The top is surmounted by a pair of elastic shoulder-braces, by which the spine is drawn back to the steel supports and thus made straight.

Fig. 3. Instrument for Backward Curvature.

Unfortunately, many of the patients with angular curvature are already fatally exhausted by caries before they see the surgeon, and cannot, in that state, tolerate the annoyance of any apparatus whatever; but, if seen in time, the above treatment, either by Fig. 1 or Fig. 3, should be promptly applied. The opinion advanced by many writers, that the spinal column should be allowed to fall forward, so as to favor anchylosis, is delusive. The periosteum will produce new bone for anchylosis quite as well in the erect position as in the crooked, besides, the falling forward keeps up the pressure, extends the caries, and insures deformity.

If the deformity to be treated is in the neck or the upper fourth of the dorsum, the before-mentioned instruments will not answer the purpose, as they do not reach high enough. In such cases it is necessary to apply the extension power to the head. For this purpose, an excellent splint may be made on the plan shown in Fig. 4. In this case we require the hip armor as in Fig. 1. From the back of this rises a steel rod to the middle of the neck, the last ten inches being in the form of a tube. In this tube slides a screw to a depth regulated by the nut upon it. The top of the screw carries a brass head-piece, hammered to a concavity to fit accurately the whole occipital region as far forward as the ears. The hollow must be lined with some soft substance, and the head kept firmly in it by a band passing around the forehead. Extension is made by turning the nut so as to cause the screw and head-piece to rise. In this way, the cervical part of the spinal column is put on stretch, and any curvature not unusually obstinate will be gradually straightened. The cost of the instrument is about $20. In case of necessity, this head extension may be added to instrument No. 1, No. 2, or No. 3.

Fig. 4. Head Extension for High Curvatures.

In adopting this treatment, the surgeon must not expect to buy his instruments ready made, and order them put on without trouble to himself. The ready made apparatus sometimes offered for sale for spinal disease always proves a miserable failure, owing to its total want of adaptation to the form of the patient.

The case should first be thoroughly investigated to ascertain what is needed. The form must then be carefully measured, and parts of it often copied in plaster of Paris, and the instrument constructed to fit under the explicit directions of the surgeon. When put on, it must not be at first screwed and buckled to the utmost tension expected to be attained, but worn lightly and easily until the skin and other parts become accustomed to its pressure. Then it may gradually be made to draw more and more until it accomplishes its purpose. Even old cases of deformity, of years standing, may thus be greatly improved and often entirely cured. The fact that the bodies of the vertebræ and the intervertebral cartilages have changed their form and become wedge-shaped, does not by any means condemn the patient to a lifelong deformity. The same agent, *pressure*, which, improperly applied, produced distortion, will, when correctly used, restore the original shape. Under the new influence, the thick sides of the bodies of the vertebræ receive the whole pressure, or, if extension is used, the shortened ligaments receive the whole tension; and, by a general law of the system, the corrected position at length becomes permanent. In some instances, the spinal column must be kept a little curved over for a time in the direction opposite to that of the deformity. The thicker borders of the vertebræ and cartilages will thus receive the entire pressure and be thinned by absorption, while the thinner sides, relieved from it, will grow thicker. In this way the forms may be quite restored.

In the same manner, the shortened muscles and ligaments of the concave side will stretch under the constant tension, and the longer ones of the opposite side contract under relaxation. The apparatus will, commonly, have to be worn from six months to two years; but after it is once well fitted, and the patient instructed in its use, the surgeon need not trouble himself by constantly watching the case. It will be better, however, for him to see it occasionally.

If circumstances permit, a system of special exercises should be adopted as an adjuvant to the other treatment. It is, however, very difficult to get your plans thoroughly carried out in this respect, unless you can have your patient an hour or two a day under the tuition and supervision of a trained assistant, or at least some resolute friend of the patient whom you have fully instructed in his duties. The limits of this essay will not permit a full description of all the movements, passive and active, which properly go to make a complete system of exercises for this disease; but a few principles may be stated, and the details must be left to the ingenuity of the practitioner:—

1st.—A few gymnastic appliances are required, such as cross-bars, cushioned posts, hand swings, etc., of sizes and forms adapted to the case. These may be erected at the patient's own house.

2d.—Examine the body of the patient critically, and determine, by experiments, what muscles would, if strengthened, tend to rectify the curvature. Such, for instance, as those along the convex sides of lateral curvatures.

3d.—Devise a system of exercises, occupying from half an hour to an hour and a-half, twice a day, which shall bring into action exclusively the muscles intended to be strengthened; but beware of mistakes, nothing is easier than for a surgeon, whose knowledge of anatomy is rusty, or his perception of mechanical relations dull, to make an erroneous plan which will bring into play the wrong set of muscles and increase the mischief.

A greatly increased development can, in time, be produced in the muscles put under training, which will powerfully assist the cure.

HIP DISEASE, (*Morbus Coxarius.*)

For the purposes of this article, hip-disease, like spinal inflammation, may be described as passing through two stages, viz.:—1st, inflammation; and, 2d, suppuration and caries. The brief intermediate stage of some authors is not, pathologically, separable from the first, in some instances, and the second in others. We observe in this, as in spinal disease, that many cases recover in the first stage without ever proceeding to caries,

The constitutional treatment for *morbus coxarius*, consists in the free employment of regimen, diet, and medicines adapted to increase the plasticity of the blood, exactly as was detailed above for spinal inflammation, bearing always in mind that *if plasticity is kept well up, caries will not occur.*

The local treatment consists, in the first stage, in the use of a suitable splint, by means of which the weight of the body and the tension of the muscles may be completely taken off from the inflamed joint. This must be accomplished by such means as will allow the patient to go about and preserve his health by exercise. The disastrous effect of the pressure and friction, produced by bearing the weight of the body upon the diseased joint, may be rendered very obvious by a few remarks. The synovial membrane, when inflamed, becomes roughened, yet upon this inflamed and rough surface the entire weight of the body presses, rubs, and grinds at every step. Of course, under such harsh usage no tissue could be expected to recover without serious mischief, and especially the exquisite machinery of a joint. The disease, therefore, being aggravated by the pressure and friction, grows daily worse, and seldom finds an interval of repose sufficiently long to permit a recovery. Hence, sooner or later, caries very commonly occurs, an abscess forms, and long and copious suppuration ensues, lasting for months and years, until the patient is exhausted and dies. In some cases, however, the endurance of the patient is so great that the carious portions of bone are actually worn to sand and washed away with the pus. In this way the head of the femur and the walls of the acetabulum may be removed, and spontaneous dislocation occur, after which, recovery takes place with a deformed hip. The part where the disease first commences is, naturally, where the pressure is greatest, viz.:—at the top of the acetabulum and

Fig. 5. Sectional View of a case of Hip-Disease.

the summit of the head of the femur. Fig. 5 is a sectional view of a case of hip-disease in a little girl 7 years of age, from whom I excised the head of the femur. The shaded portion represents a mass of necrosed fragments which had been originally parts of the wall of the acetabulum. The black spot above is a fistulous channel in the bone through which the pus made its escape. The head of the femur is seen roughened and worn to a stump by constant attrition against the dead fragments of bone. After the removal of the diseased bone, the patient recovered rapidly, and now walks on the limb with ease,—a ligamentous attachment of the femur to the pelvis supplying the place of the lost joint.

The local treatment of hip-disease, in the first or inflammatory stage, consists in the application of some suitable instrument, by which the weight of the body and the tension of the muscles can be entirely taken off from the joint, so that the inflamed surfaces no longer press and rub against each other. Dr. H. G. Davis, of New York, was the first to construct an efficient apparatus for this purpose, and with it he has accomplished many excellent cures. There are some defects, however, in the practical working of his instrument, which have led me to devise a modification, which, after much experience in these cases, I prefer. It is represented in Fig. 6, and consists of the following parts:—1st, an iron crutch-piece modelled accurately to fit the perineum and nates. The engraving conveys an erroneous idea about the shape of this part. The principal curve is lateral, so as to embrace half the circumference of the thigh at the level of the fold of the nates.

Fig 6.

Splint for Hip-Disease.

The posterior extremity is broad and hollowed to fit the nates, so that the patient, as it were, sits upon it. It is cushioned and covered with enamelled cloth or patent leather, to resist the moisture of the perspiration. The crutch piece thus made is supported upon the summit of a strong screw, twelve inches in length, upon which turn two octagonal nuts. The screw slides into a tube, and this again terminates at its lower extremity in a rod which runs down along the inner side of the leg to the ground, and, by a cross-piece, rivets firmly to the sole of a stout shoe. The top of the shoe carries a light buckle on either side for the purpose hereafter mentioned.

The instrument is applied to the patient as follows:—Place it on the inner side of the limb, in such a position that the crutch-piece will press upward against the perineum, the broad end being backwards. The concave edge will now embrace about half the circumference of the thigh, and the perineum and nates will rest easily in the hollow of the upper surface. Buckle the attached strap lightly around the outer side of the thigh. Next cut two adhesive-straps, each two feet in length, and three inches wide at the one end and one at the other; apply these on each side of the limb, broad end upwards, and confine them by winding spiral straps over them as in adhesive-strap extension for fractures. Place the foot in the shoe, and the lower ends of the adhesive-straps in the buckles at the top of it. Tighten the straps in the buckles until the foot rests firmly in the bottom of the shoe. Next extend the screw, by turning the nuts, until the crutch-piece rests firmly against the perineum, and until the patient, in walking, bears all his weight on the instrument and *none of it* on the hip-joint. This can be ascertained by seeing if the adhesive-straps are still tense when the weight of the body is thrown upon the instrument. The patient may then be allowed to walk about as much as he pleases, preserving his general health by exercise. He will not require any crutches. It should have been observed, that it is best to have two nuts upon the screw. When the lower one is set at the right length, it should be held firmly while the upper one is screwed strongly down against it. This is simply the common device of machinists to fix a nut in a stationary position.

The two will then stand immovable without working up or down. In the use of this instrument, the patient is soon conscious of great relief. Even little children discover in a few days that it greatly relieves their pain, and insist upon keeping it on. It should be worn nights as well as daytimes, except in the milder cases. From the hour of its application, the patient generally begins to improve, and by degrees is perfectly cured. He should wear the splint from six months to two years. The cost of the instrument is $15.

In cases where the thigh has been drawn up at a right angle with the body, by the contraction of the flexors, it is sometimes necessary to divide the tendons and bring down the limb before the splint can be usefully worn.

The second stage of hip-disease is that of caries. When this has occurred, a recovery by simple subsidence of inflammation is no longer possible. The dead bone must be extruded by nature or removed by the surgeon. Great fear was formerly felt of undertaking an operation for this purpose, and the books which condemn it are still standard works. There is, however, no part of the body whatever more benefited by the removal of carious bone than the hip-joint. The operation is not particularly dangerous, and has saved many lives. It is best performed by a single straight incision along the trochanter major, through which the head of the femur may be turned out and sawn off. If the ilium is carious, it must be freely and unhesitatingly trimmed with the gouge until all dead portions are removed.

Fig. 7. Apparatus for Straightening Flexed Hips.

After the operation, as before, the splint must be worn to keep the limb from shortening, until the femur has had time to contract a ligamentous adhesion to the ilium.

We often meet old cases of hip-disease, in which the carious bone has already been removed by exfoilation, and the ulcerations have healed up; or, perhaps, the inflammation has subsided without producing caries, but, owing to mismanagement during the convalescence, the thigh has become stiffened in the flexed condition, so that the foot cannot be brought down to the ground. In these cases, the hip-disease proper is already cured, and we have only to deal with the deformity, and if we succeed in rectifying that, so as to bring the foot to the ground, we shall give the patient a useful limb.

For this purpose, I employ the apparatus shown in Fig. 7. It consists of a pair of strong close-fitting drawers, made of double brown drilling, and extending from the top of the abdomen to the knee. Enclosed in this is a brass curiass fitted to the front of the abdomen and pelvis, and a brass armor covering the front half of the thigh. The top of the thigh-piece is solidly hinged to the lower edge of the curiass. From one piece to the other passes an extension-brace, which, when the screw is turned, slowly pushes down the thigh and corrects the deformity. A slight additional extension should be made every day until the object is accomplished. The cost of the instrument is $10.

### KNEE-JOINT DISEASE.

The knee-joint is subject to precisely the same inflammatory and carious affections as the hip and the spine. The only notable difference in their history is, that hip-disease is limited almost exclusively to children, while spinal and knee-diseases occur at every age. The pathology of knee-disease is identical with that of the hip, and the tendency of carious spots, in one of the bones, to produce caries in the corresponding spot of the bone which rests upon it, is still more obvious than in the hip. Fig. 8 is a vertical plan of a knee-joint which I removed by the operation of resection; and the shaded portions represent the dead part of the bone. It is curious to note how exactly each sequestrum is matched by another of the same size and position, facing it from the opposite surface of the joint. The two sequestra in the femur were, probably, first formed, and by the constant irritation which they kept up, they caused the death of those spots in the patella and tibia which rested upon them.

It is noteworthy also that the disease has occurred exactly at the two points which are subjected to the greatest pressure in the use of the limb.

I have introduced this engraving for the express purpose of showing the injurious effects of pressure, and of impressing upon the reader the importance of removing that cause of evil by suitable extension splints. The treatment, therefore is identical with that of hip-disease, and the same instrument (see Fig. 6,) is required. The uniform conclusion, from the best experience is, that this treatmemt, applied in the first stage, is even more successful in the knee than in the hip. If, however, the case has already proceeded to the stage of caries, the splint is no longer applicable. There should then be an early resort to resection or amputation, before the patient is worn out by suppuration and pain.

Fig. 8. Vertical Section of a Knee-Joint, showing that Necrosis in one surface causes Necrosis in the surface in contact with it.

CLUB-FOOT, (*Talipes.*)

The recent improvements in mechanical surgery bid fair to abolish almost entirely the operation of cutting the tendons in club-feet. Some very excellent surgeons, both in this country and in England, now treat this deformity almost wholly without tenotomy, it being found that the contracted tissues will always yield to a steady tension properly applied. Even old and apparently hopeless dislocations of the hip-joint have yielded to steady tension kept up for weeks by means of elastic bands, so that the head of the bone was gradually brought down and replaced in the socket.

*Every distorted joint may be made to return to its normal position, by steady and long continued traction.* The principle of the management of talipes without tenotomy is, therefore, very simple; but the successful application of it depends upon the patience, faithfulness, and ingenuity of the surgeon. There are also a few instances where the practical difficulties render the principle inapplicable. The appliances must be prepared by the surgeon for each particular patient, and varied to suit the pecularities of the case; and the materials for them consist mainly of adhesive-plaster and elastic webbing. The following description may serve to convey the general idea. We will suppose it to be a case of talipes varus. The first thing to be done is to secure two firm points of traction, which will not hurt the patient. For the first, we envelope the foot in bands of adhesive-plaster, carefully adjusted, bringing their free ends under the sole and up the outer side. They are there gathered in one, two, or three groups, or sometimes all attached to a small rod running parallel to the outer border of the foot. The second point of tension is easily made by attaching broad adhesive-straps to the upper part of the outer side of the leg. It is convenient to arm the lower extremities of these with light buckles. The upper and lower adhesive-straps are now connected by from one to three strips of elastic webbing which, of course, pass over the outer maleolus and tend to draw the foot to its position. A small cushion should be placed upon the maleolar region to receive the pressure of the bands. Thus prepared, let the elastics be buckled to a very gentle tension for the first few days, until the skin becomes accustomed to the presence of the apparatus, after which, they may be gradually tightened. The tension being moderately kept up day and night occasions very little pain, and the contracted parts slowly yield until the foot assumes a perfect position. Many weeks are often consumed in the treatment; but if the parents are intelligent, the surgeon need not see the child very often after the first twelve days.

Many other applications of these principles will readily suggest themselves to the ingenious practitioner, but which cannot be detailed in this brief essay.

We may truly say that, for those afflicted with spinal curvature, hip-disease, inflammation of the knee, or club foot, a new era has dawned; and vast numbers of cases supposed by our predecessors to be hopeless, will, in our day, be restored to soundness and perfect form.

## Figures and Tables

**Fig. 1. f1:**
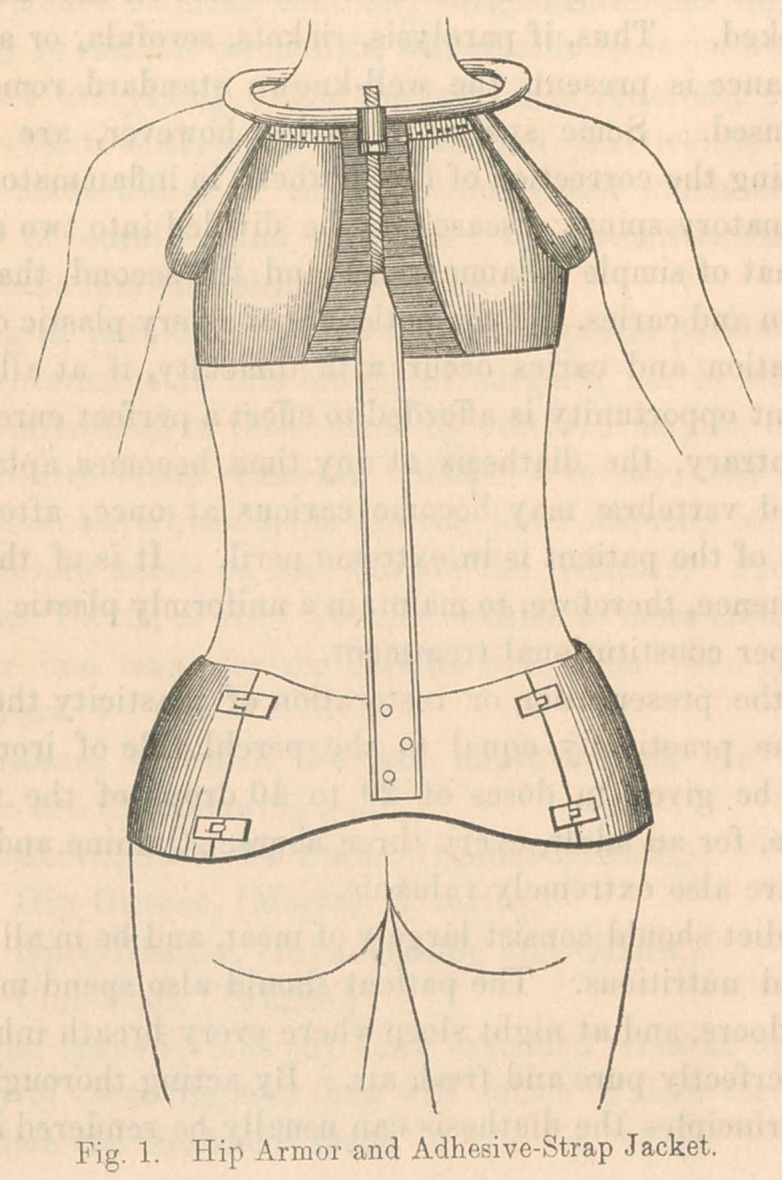


**Fig. 2. f2:**
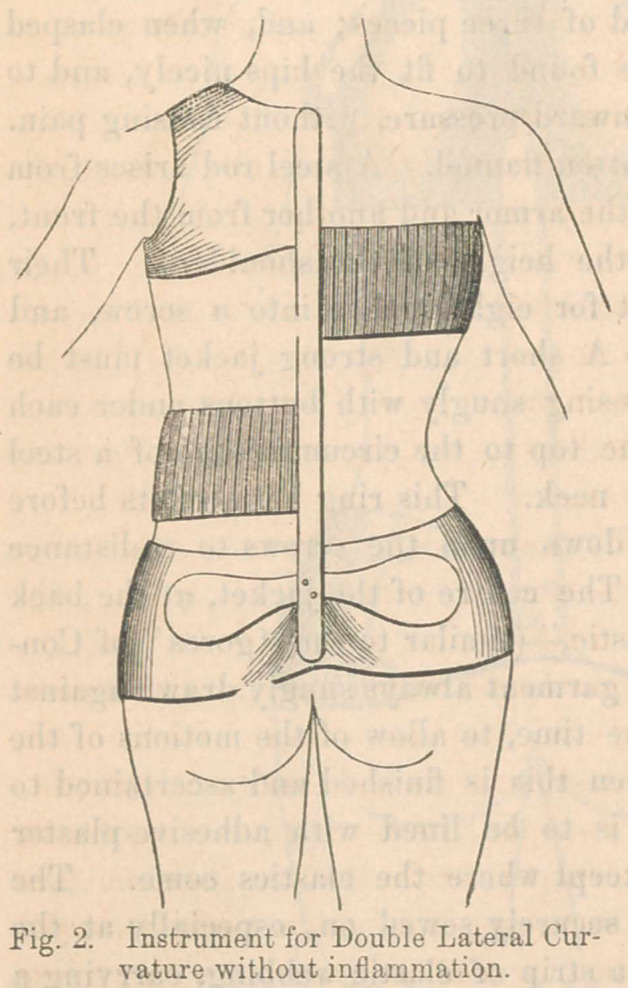


**Fig. 3. f3:**
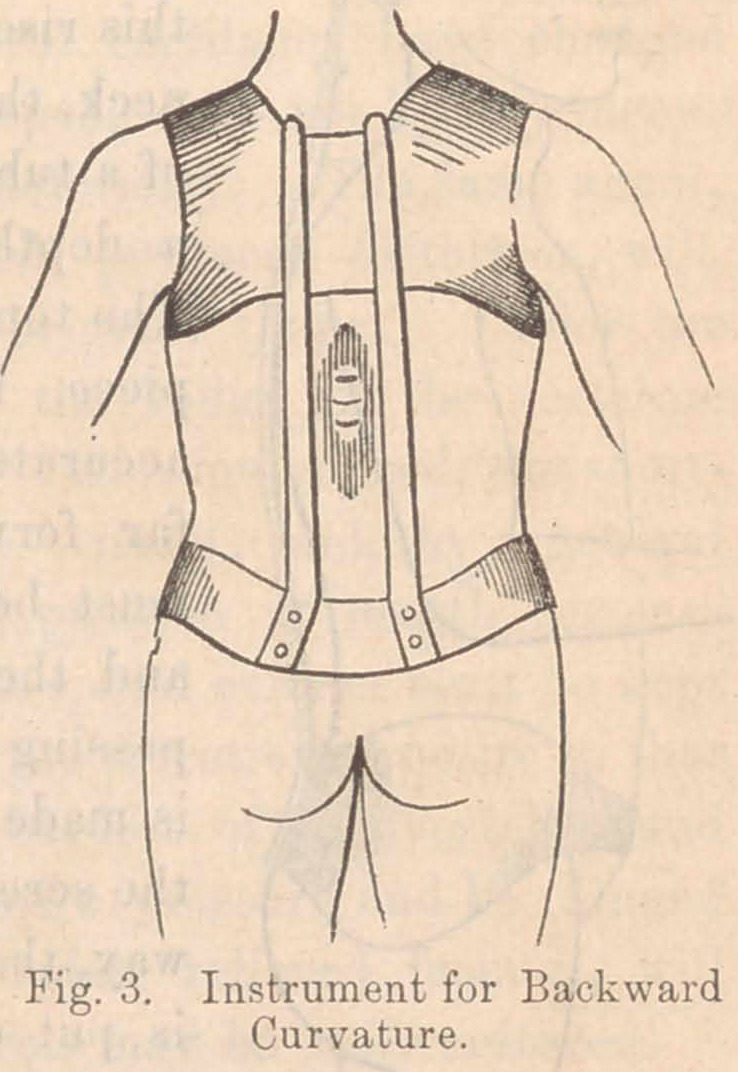


**Fig. 4. f4:**
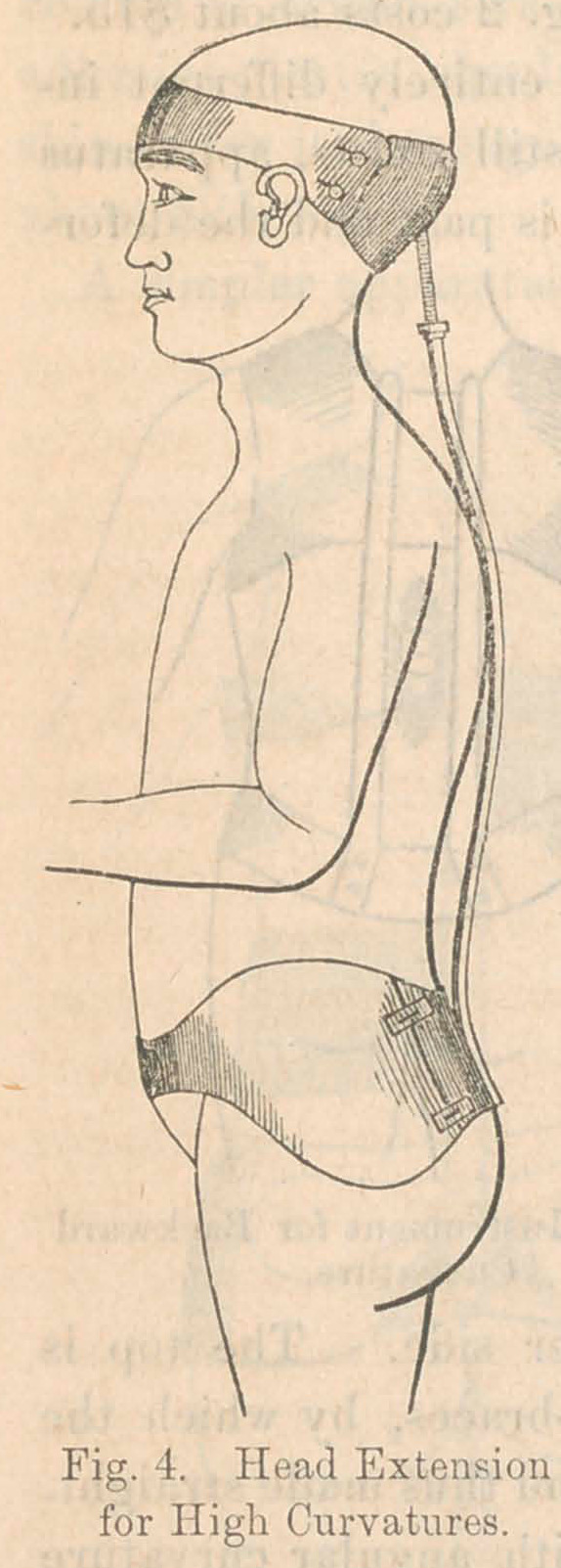


**Fig. 5. f5:**
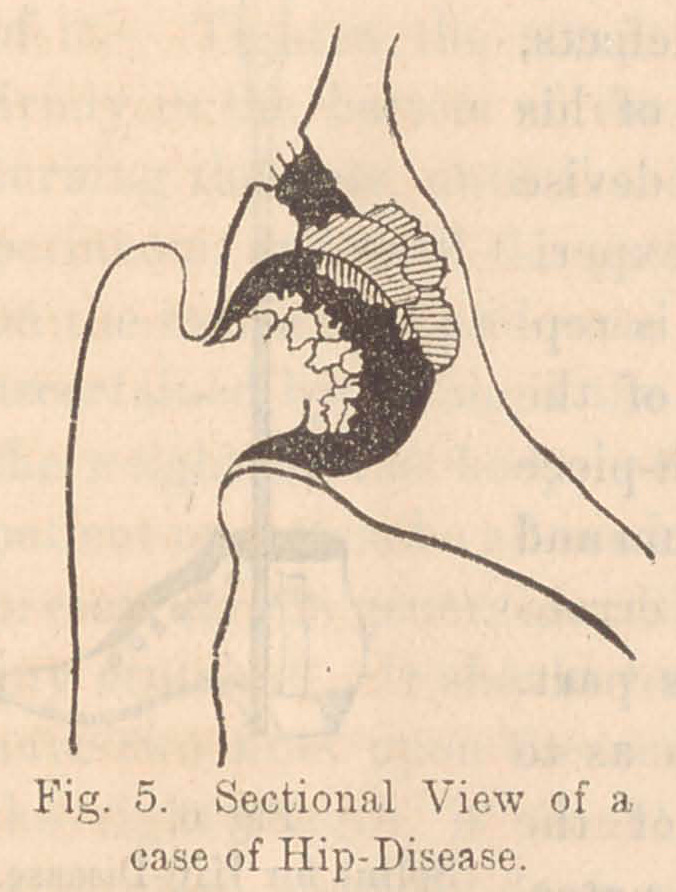


**Fig. 6. f6:**
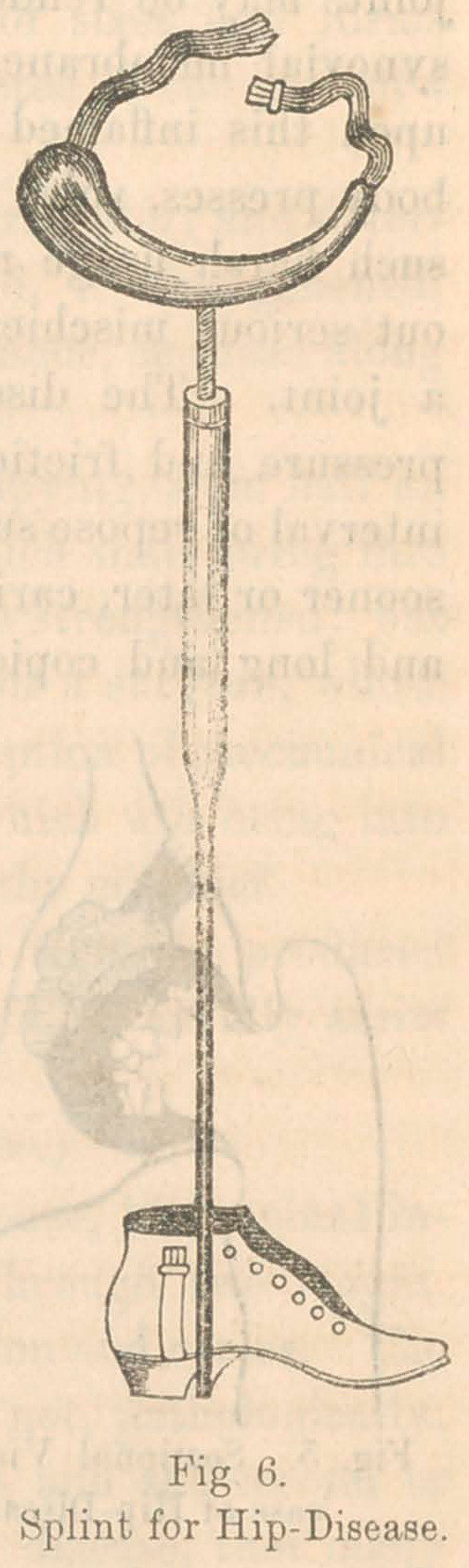


**Fig. 7. f7:**
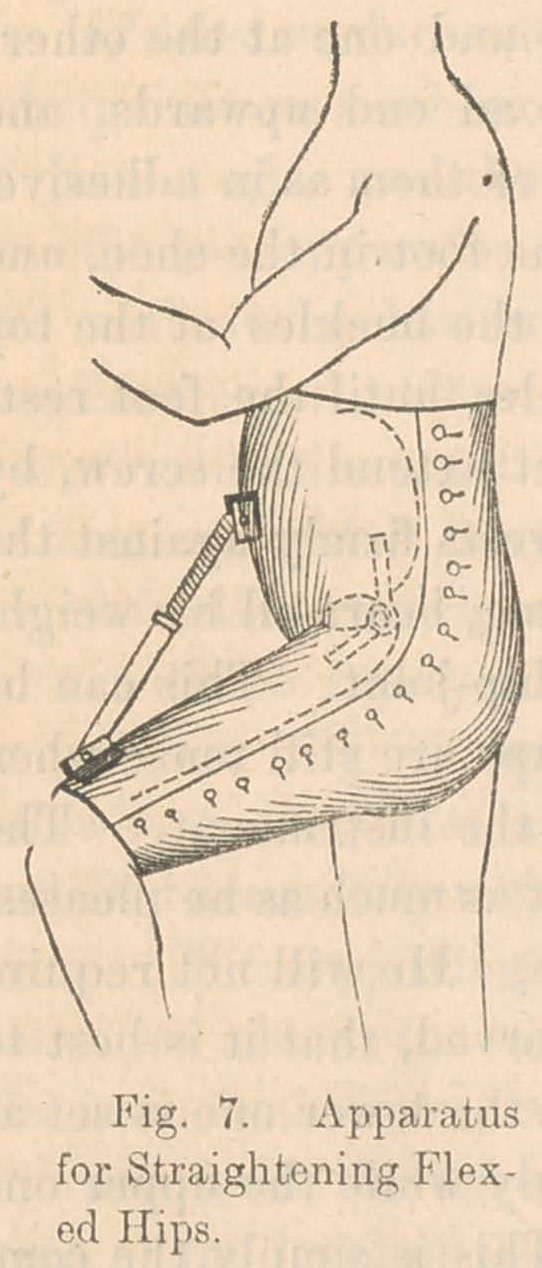


**Fig. 8. f8:**